# *foxm1* Modulates Cell Non-Autonomous Response in Zebrafish Skeletal Muscle Homeostasis

**DOI:** 10.3390/cells10051241

**Published:** 2021-05-18

**Authors:** Fábio J. Ferreira, Leonor Carvalho, Elsa Logarinho, José Bessa

**Affiliations:** 1 i3S—Instituto de Investigação e Inovação em Saúde, Universidade do Porto, 4200-135 Porto, Portugal; fabio.ferreira@i3s.up.pt (F.J.F.); leonor.carvalho411@gmail.com (L.C.); 2 Vertebrate Development and Regeneration Group, IBMC—Instituto de Biologia Molecular e Celular, Universidade do Porto, 4200-135 Porto, Portugal; 3 Aging and Aneuploidy Group, IBMC—Instituto de Biologia Molecular e Celular, Universidade do Porto, 4200-135 Porto, Portugal; 4 Graduate Program in Areas of Basic and Applied Biology (GABBA), Instituto de Ciências Biomédicas Abel Salazar (ICBAS), Universidade do Porto, 4050-313 Porto, Portugal; 5 Departamento de Biologia Animal, Faculdade de Ciências, Universidade de Lisboa, 1749-016 Lisboa, Portugal

**Keywords:** zebrafish, *foxm1*, CRISPR/Cas9, skeletal muscle, satellite cells

## Abstract

*foxm1* is a master regulator of the cell cycle, contributing to cell proliferation. Recent data have shown that this transcription factor also modulates gene networks associated with other cellular mechanisms, suggesting non-proliferative functions that remain largely unexplored. In this study, we used CRISPR/Cas9 to disrupt *foxm1* in the zebrafish terminally differentiated fast-twitching muscle cells. *foxm1* genomic disruption increased myofiber death and clearance. Interestingly, this contributed to non-autonomous satellite cell activation and proliferation. Moreover, we observed that Cas9 expression alone was strongly deleterious to muscle cells. Our report shows that *foxm1* modulates a muscle non-autonomous response to myofiber death and highlights underreported toxicity to high expression of Cas9 in vivo.

## 1. Introduction

FOXM1 is a transcription factor considered a master regulator of the cell cycle since it promotes expression of the late cell cycle gene cluster [1,2,3]. Mammalian FOXM1 expression is increased in proliferative cells and is commonly found in processes involving cell division, such as embryogenesis, tissue repair and cancer [4,5,6,7]. Null mice for *Foxm1* exhibit abnormalities in multiple organs concomitant with embryonic lethality [8,9,10]. It has been reported that while cardiomyocytes and hepatoblasts fail to complete mitosis, other cell types exhibit no visible change in proliferation rate in *Foxm1* knockout mouse embryos, which suggests cell-specific functions [5]. Knockdown of *foxm1* with high doses of morpholino is lethal in zebrafish embryos [11], while the cell cycle role of *foxm1* in zebrafish tissue regeneration has also been particularly explored [12,13]. Mammalian and zebrafish FoxM1 homologues share the main features of protein structure and active domains [14], namely the N-terminal negative regulatory domain (NRD), which is responsible for autoinhibitory activity [15,16], the DNA binding domain (DBD) and the C-terminal transactivation domain (TAD), which contains several post-translational modification sites and recruits the transcriptional machinery to target regulatory elements [17,18]. Loss of the C-terminal domain of FoxM1 results in downregulation of its target genes [19].

Recent data have shown that FOXM1 also regulates genes involved in many biological processes beyond cell cycle, such as DNA damage repair and cytokine production, namely in response to aging-associated cell senescence [3,20]. FOXM1 also appears to be required for mitochondrial homeostasis [21] and proper cell differentiation [5]. These results suggest that FOXM1 might have a role beyond its canonical function in proliferative cells, although its role in non-proliferative tissues has not yet been extensively explored.

Degenerative loss of skeletal muscle homeostasis with age occurs in parallel with organismal decrease of FOXM1 expression in humans [3], leading to the question of whether the former may be, in part, consequence of the latter. Crucially, high conservation of muscle gene networks and structure between mammals and zebrafish have been described [22,23]. In zebrafish, fast-twitching fibers become functional around 24 h post fertilization (hpf), when they can be found in the deep portion of the myotome and the compartmentalization of the zebrafish somites is complete [24,25]. From then on, secondary myogenesis supports the indeterminate growth of zebrafish through a balance between self-renewal of the resident stem cells, the Pax7-positive satellite cells, and cell differentiation [26,27]. Muscle grows through the combined increase in fiber size, resulting from fusion of myogenic progenitors to the fibers, and number, by satellite cells differentiation into new myofibers [25]. Zebrafish muscle has been previously used as a model to study muscle disease [28,29,30] and response to injury [31,32]. Upon injury, quiescent satellite cells in the myotome are activated and divide asymmetrically, resulting in renewal of the stem cell niche and the generation of new muscle fibers [27]. Mouse satellite cells express *Foxm1*, which contributes to proliferation and survival of these stem cells and proper muscle regeneration upon injury [33,34]. However, the high levels of *Foxm1* expression in satellite cells decrease substantially, albeit not totally, upon differentiation into myofibers [33]. Similarly, mouse myofibers express very low levels of *Foxm1* [35]. Yet, it remains unknown whether *foxm1* is required for proper cell function in zebrafish myofibers.

CRISPR/Cas9 technology has been used to study gene function and disease in zebrafish [36]. CRISPR/Cas9-mediated genome editing relies, in part, on the error-prone non-homologous DNA end joining (NHEJ) mechanism to repair DNA double-strand breaks (DSBs), which results in small de novo insertions and deletions (indels) [37]. Indels can then result in frameshift and non-sense mutations, with the potential to knockout gene expression either transiently, in injected F0 animals (crispants), or stably, in F1 and F2 animals [36,38,39]. More recent applications of CRISPR/Cas9 in zebrafish have shown that using tissue-specific expression of Cas9, it is possible to target genes in a tissue-specific manner [40]. Notwithstanding the revolutionary usefulness of CRISPR/Cas9 technology in biomedical research, recent studies have highlighted potential issues with high levels of DSBs induced by Cas9 [41,42].

In this study, we explored the role of *foxm1* in non-proliferating differentiated cells to better dissect its function beyond cell cycle progression, using zebrafish. Advantages of this animal model stand from the high number of embryos generated in each crossing, which are amenable to genetic manipulation by injection in one-cell stage, are fully transparent and develop rapidly ex utero. Our initial attempts to generate *foxm1* mutants suggest early embryonic lethality. To bypass this problem, and in order to understand the function of *foxm1* in post-mitotic cells, we used the zebrafish embryo fast-twitch muscle fibers as a model since they become functional around 24 hpf, can be promptly genetically manipulated and are easily trackable in vivo. Driving tissue-specific expression of Cas9 in fast-twitch muscle fibers to target *foxm1*, we found that overexpression of Cas9 alone in these myofibers induces apoptosis and cell number decline. In this context, upon *foxm1* loss-of-function in fast-twitch muscle fibers, we found that *foxm1* contributes to cell communication and non-autonomous satellite cell activation in response to Cas9-induced cell injuries. These findings support previous data suggesting FoxM1 as a regulator of DNA damage, cytokine secretion and cell communication. Our results also highlight the impact of exogenous Cas9 overexpression in cell viability, raising concerns about cell competition and tissue repair mechanisms that may hinder in vivo CRISPR/Cas9-mediated mutagenesis approaches.

## 2. Materials and Methods

### 2.1. Zebrafish Maintenance

Adult wild-type Tuebingen (TU) zebrafish (*Danio rerio*) were maintained in a recirculating system under conditions approved by the i3S Animal Welfare and Ethics Committee and the Portuguese National Authority for Animal Health (DGAV). Zebrafish embryos and larvae were reared in incubators at 28 °C during experiments or until approximately 7 days post fertilization (dpf), being then transferred to a recirculating system.

### 2.2. Production of sgRNA and Cas9 mRNA

Nine sgRNA spacer sequences targeting *foxm1* coding sequence were selected on Benchling (2018) [43] based on metrics from Doench and colleagues [44] and Hsu and colleagues [45]. Pairs of oligonucleotides where ordered (Sigma-Aldrich, Darmstadt, Germany) and annealed, followed by cloning in the BsaI-digested vector pDR274 (Addgene #42250), as described [46]. sgRNAs were transcribed using the T7 RNA polymerase (Thermo Scientific, Vilnius, Lithuania) and the HindIII-digested sgRNA expression vector as template. The Cas9 mRNA was transcribed using the SP6 RNA polymerase (Thermo Scientific, Vilnius, Lithuania) and the NotI-digested pCS2-nCas9n (Addgene #47929) as a template, and G(5′)ppp(5′)G RNA Cap Structure Analog (New England Biolabs, Ipswich, MA, USA). The transcribed RNAs were purified using a Sephadex column, followed by the phenol-chloroform extraction [47], prior to injection. sgRNA sequences can be found in the Table S1.

### 2.3. Microinjection in Zebrafish Embryos

sgRNA and Cas9-encoding mRNA were coinjected into the cell of one-cell stage zebrafish embryos. Each embryo was injected with approximately 5 nL of a mixture containing 150 ng/μL of sgRNA, 200 ng/μL of Cas9 mRNA and 10% of phenol-red. Embryos were placed in Petri dishes with E3 medium supplemented with N-phenylthiourea, in batches of 50 embryos per plate, and reared at 28 °C.

### 2.4. Detection of Mutations

At about 24 hpf, genomic DNA was extracted from 3 pools of 8 embryos and the targeted genomic locus was amplified with the primers presented in the Table S2. The product was then denatured and reannealed to promote the formation of heteroduplexes. The presence of heteroduplexes was then assessed with an 8% polyacrylamide gel electrophoresis (PAGE). Selected bands were cut, reamplified and sent for Sanger sequencing in i3S Genomics Scientific Platform. Sequences from at least 3 different embryo batches were analyzed with TIDE [48]. Graphs with data derived from TIDE report the percentage of indels statistically significant (*p* value < 0.05). Percentages are based on the analysis of a 20-nucleotides window centered in the Cas9 cutting site. Cell type-specific CRISPR/Cas9-mediated targeting of *foxm1* using mylfpa:Cas9-T2A-GFP;U68.2 (see Section 2.9 of Materials and Methods) was analyzed with DNA extracted from 2 dpf single embryos and DNA extracted from FACS-sorted GFP-positive cells from 2 dpf embryos.

### 2.5. Identification of Founders and Stable Mutant Lines

Animals injected with sgRNA were reared until 3-month-old, at which point they were outcrossed. Genomic DNA was extracted from 3 pools of 8 embryos and processed as described above. Animals transmitting mutations in the targeted region of *foxm1* (F0) were considered founders and their offspring (F1) was reared until 3-month-old. These F1 animals where then genotyped for the mutation and used to initiate stable mutant lines, with well-characterized mutations.

### 2.6. Fluorescence-Activated Cell Sorting (FACS)

Groups of 400 DsRed-positive 3 dpf embryos were selected and dechorionated. After washing with 1× PBS, embryos were digested at 32 °C with 0.125 mg/mL collagenase II in HBSS (Ca^2+^ Mg^2+^, 1 mM CaCl_2_) under 800 rpm shaking for 30 min and mechanical dissociation every 5 min using a micropipette with low adhesion tips. The cell mixture was centrifuged for 10 min at 500 × *g* at 4 °C and the pellet washed with 1× PBS. The cells were then resuspended in sorting buffer (1× PBS, 0.5% BSA, 0.5 M EDTA, 1 M HEPES) filtered with a 40 µm mesh cell strainer and kept on ice until sorting. Cell sorting was performed in a BD FACSAria II flow cytometer (BD Biosciences, San Diego, CA, USA) using the yellow/green laser (561 nm). Cells were gated by forward scatter area (FSC-A) vs. side scatter area (SSA-A) and FSC-A vs. FSC-height (FCS-H) plots to exclude dead cells and doublets/clumps, respectively. The gates were established based on the autofluorescence of WT embryo cells. Cells were selected based on the DsRed expression. The collected cell suspension was centrifuged at 300 × *g* for 10 min and the pellet was resuspended in TRIzol (Ambion, Life Technologies, Carlsbad, CA, USA), vortexed and stored at −20 °C. The same procedure was applied to groups of 50–100 GFP-positive 2 dpf embryos injected with mylfpa:Cas9-T2A-GFP;U6:8.2, but the blue laser (488 nm) was used and the GFP-positive sorted cells were collected to 1× PBS and further used for genotyping.

### 2.7. Tissue-Specific CRISPR/Cas9

The MultiSite Gateway technology was used to create vectors driving expression of Cas9 specifically in muscle cells. Briefly, a 1999 bp sequence of the mylfpa promoter cloned in the p5E-MCS (Addgene #26029), kindly provided by the David Langenau’s group, the pME-Cas9-T2A-GFP (Addgene #63155) and the p3E-polyA (Tol2kit v1.2 #302) were cloned in the pDestTol2pA2-U6:sgRNA (Addgene #63157) using the LR Clonase II Plus enzyme (Invitrogen, Bleiswijk, Netherlands). The functional sgRNAs targeting *foxm1* was then cloned in the BseRI-digested final vector (Addgene #63157). A version of the final vector without any introduced sgRNA was used as a negative control. To follow *mylfpa*-positive cells in the absence of Cas9 expression, the pME-mCherry (Tol2kit v1.2 #386) was used as a middle entry vector instead of pME-Cas9-T2A-GFP and no sgRNA was inserted in the final vector.

### 2.8. Tol2 Transposase Synthesis

The Tol2 transposase mRNA was transcribed using the SP6 RNA polymerase (Thermo Scientific, Vilnius, Lithuania) and the NotI-digested pCS2FA-transposase vector (Tol2kit v1.2 #396), and including G(5’)ppp(5’)G RNA Cap Structure Analog (New England Biolabs, Ipswich, MA, USA), as described [47]. The transcribed RNAs were purified as described above.

### 2.9. Tissue-Specific Mutagenesis Assay

One-cell stage embryos were injected with approximately 5 nL of a mixture containing 25 ng/μL of Tol2 transposase mRNA, 50 ng/μL of the transgenesis-control vector and 150 ng/μL of either sgRNA containing vector or the empty vector. At 1, 2, 3 and 5 dpf embryos were screened under a fluorescence stereomicroscope (Leica M205, Leica Microsystems, Wetzlar, Germany) and photographed (Hamamatsu ORCA-Flash4.0 LT camera, Hamamatsu Photonics, Hamamatsu, Japan). GFP-positive and mCherry-positive cells were quantified at each timepoint, using Fiji-ImageJ [49]. The total number of cells in each embryo was normalized to the average number of cells in embryos from the control sample.

### 2.10. RNA Extraction and Reverse Transcription

RNA was extracted from FACS-sorted cells and 3 dpf embryos with TRIzol (Ambion, Life Technologies, Carlsbad, CA, USA), following the manufacturer’s instructions. RNA was treated with DNase I (Thermo Scientific, Vilnius, Lithuania). Samples were quantified in NanoDrop 1000 and stored at −80 °C. RNA was retrotranscribed into cDNA using the SuperScript II Reverse Transcriptase (Invitrogen, Carlsbad, CA, USA), according to the manufacturer’s instructions.

### 2.11. Real Time Quantitative PCR (RT-qPCR)

RT-qPCR was performed using iTaq Universal SYBR Green Supermix (Bio-Rad, Hercules, CA, USA) in a thermocycler (CFX96 Touch Real-Time PCR Detection System, Bio-Rad, Hercules, CA, USA) with the following program: 95 °C for 3 min followed by 39 cycles of 95 °C for 30 s, 56 °C for 30 s and 72 °C for 30 s. Non-reverse transcribed and blank controls were included. Six biological replicates with three technical replicates were used per target gene. Expression was normalized with the *b2m* and *eef1a1* housekeeping genes, and different biological replicates were normalized to the mean expression of the control (DsRed-negative cells). Primers used in RT-qPCR are presented in Table S3.

### 2.12. Cleaved Caspase-3 Immunostaining and Quantification

Cleaved caspase-3 immunostaining was performed as previously described [50], with minor adjustments. Briefly, embryos injected with mylfpa:mCherry, mylfpa:Cas9-T2A-GFP or mylfpa:Cas9-T2A-GFP;U6:8.2, and non-injected embryos, were sacrificed with MS-222 overdose at 2 dpf and fixed in 4% paraformaldehyde (Electron Microscopy Sciences, Hatfield, PA, USA) in phosphate-buffered saline (1× PBS) overnight at 4 °C. Embryos were permeabilized with 100% methanol at −20 °C for 2 h to 24 h. After rehydration in 75%, 50% and 25% methanol in PDT (1× PBS, 0.1% Tween-20, 0.3% Triton-X, 1% DMSO), embryos were washed twice in PDT for 30 min at RT and incubated in blocking buffer (1× PDT, 10% heat-inactivated fetal bovine serum, 2% bovine serum albumin) for 1 h at RT. Embryos were then incubated overnight with an anti-cleaved caspase-3 antibody (1:200, Cat. PC679, Calbiochem, Merck, Darmstadt, Germany) diluted in blocking buffer. After washes in PDT, embryos were incubated with anti-rabbit Alexa 488 (1:800, Invitrogen, Eugene, OR, USA) or anti-rabbit Alexa 568 (1:800, Invitrogen, Eugene, OR, USA) diluted in blocking buffer for 4 h at RT. Embryos were washed in PDT as above and stored at 4 °C in 50% glycerol in 1× PBS. Microscopy slides were prepared using 50% glycerol in 1× PBS. Z-stacks of the embryo’s trucks were obtained with a fluorescence stereomicroscope (Leica M205, Leica Microsystems, Wetzlar, Germany) and camera (Hamamatsu ORCA-Flash4.0 LT, Hamamatsu Photonics, Hamamatsu, Japan). Cleaved caspase-3-positive myofibers cells were quantified using Fiji-ImageJ [49].

### 2.13. Pax7 Immunostaining and Quantification

Embryos injected with mylfpa:Cas9-T2A-GFP or mylfpa:Cas9-T2A-GFP;U6:8.2 were sacrificed with MS-222 overdose and fixed in 4% paraformaldehyde (Electron Microscopy Sciences, USA) in phosphate-buffered saline (1× PBS) overnight at 4 °C, rinsed in 0.1% PBS-T (0.1% Triton X-100 in 1× PBS), permeabilized 1 h with 0.5% PBS-T and blocked 1 h with blocking buffer (2% normal goat serum, 2 mg/mL BSA, 0.1% Tween20 in PBS). Embryos were incubated overnight with an anti-Pax7 antibody [51] (1:50, monoclonal, Developmental Studies Hybridoma Bank, Iowa City, IA, USA) diluted in blocking buffer. Embryos were then incubated with anti-mouse Alexa 568 (1:800, Invitrogen, Eugene, OR, USA) and DAPI (1:1000, Invitrogen, Eugene, OR, USA), diluted in blocking buffer. Embryos were rinsed as above and stored in 50% glycerol in 1× PBS. Microscopy slides were prepared using 50% glycerol in 1× PBS. Images were acquired with a laser point scanning confocal microscope (Leica TCS SP5, Leica Microsystems, Wetzlar, Germany). Positive satellite cells in the central myotome and the vertical and horizontal myosepta where quantified, while xanthophores, pigment cells in the surface of somites, with higher intensity staining and bean-shaped nuclei were excluded [52].

### 2.14. Statistical Analysis

The statistical analysis was performed using Microsoft Office Excel and GraphPad Prism 8 software. Significant differences (*p* value < 0.05) from 6 independent experiments in the qPCR assay and 13 embryos in the Pax7 immunostaining assay were determined by a Student’s *t*-test. Significant differences (*p* value < 0.05) between at least 34 embryos per conditions in the tissue-specific mutagenesis assay were calculated with a one-way ANOVA with Bonferroni correction for multiple comparisons. Significant differences (*p* value < 0.05) between samples with ≥42 embryos per condition in the cleaved caspase-3 immunostaining assay were determined by the Fisher’s exact test using the discrete values.

## 3. Results and Discussion

### 3.1. foxm1 Loss-of-Function Is Deleterious during Zebrafish Embryogenesis

**Figure 1:**
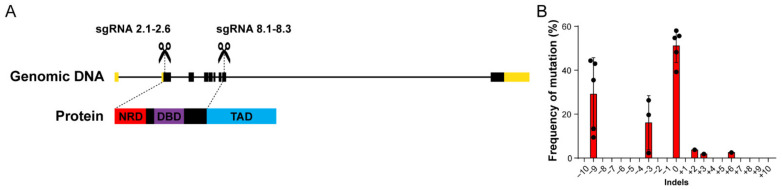
*foxm1* CRISPR/Cas9-mediated disruption results in in-frame deletions. (**A**) The genomic sequence of *foxm1* highlighting the 5′ and 3′ UTRs (yellow boxes), exons (black boxes) and regions targeted with CRISPR/Cas9. This strategy targets the NRD and the TAD domains. (**B**) Statistically significant deletions (*p* value < 0.05) detected at 24 hpf in batches of 8 embryos injected with sgRNA 8.2. Frequency reflects abundancy of WT and mutant alleles. *n* = 5.

CRISPR/Cas9 technology can be used to produce frameshift-inducing indels in zebrafish embryos [53]. Frameshift mutations can lead to mRNAs with premature termination codons (PTCs) or mRNAs coding aberrant, non-functional proteins. PTCs often result in nonsense-mediated decay (NMD) of the target transcript, with consequential genetic knockdown [54,55]. In zebrafish, the NMD effectors are transmitted maternally and expressed ubiquitously from early embryogenesis [56]. With that in mind, we designed six sgRNAs targeting the exon 2 of *foxm1* (sgRNAs 2.1–2.6), downstream the start codon, to generate a PTC that would lead to *foxm1* knockdown in zebrafish (Figure 1A). We also designed three sgRNAs targeting exon 8 (sgRNAs 8.1–8.3), aiming to disrupt the sequence coding the protein transactivation domain (TAD) (Figure 1A). Since all possible premature out-of-frame stop codons created by targeting exon 8 of *foxm1* are downstream the last exon junction, NMD is unlikely to function efficiently [56,57], meaning frameshift mutations may ultimately result in an unstable mRNA or protein that lacks its C-terminal TAD. In fact, in humans an alternatively spliced FOXM1 transcript generates a frameshift and a PTC in the C-terminal domain that results in a truncated protein with dominant negative activity [19]. Moreover, indels that keep the correct transcription reading frame may still affect protein function by affecting post-translational modification motifs of *foxm1* in the targeted region [18,58,59]. In each experiment, we confirmed the efficiency of CRISPR/Cas9 by knocking down expression of eGFP in a stable line previously generated in the lab (*Tg(elavl3:GFP)*), using the same Cas9 mRNA batch (Figure S1A–C). A PAGE-based assay to detect heteroduplexes [60] revealed the presence of indels only in the target site of sgRNA 8.2 out of the nine tested sgRNAs (Figure S1D–G). Our results contrast with previous reports showing a higher mutagenesis efficiency for different sgRNAs in zebrafish [46]. These results suggest that most indels in the zebrafish *foxm1* sequence are strongly deleterious leading to a selection of non-mutated cells. Additionally, these data suggest that loss of *foxm1* is not transcriptionally compensated by other genes [61], such as other fox genes, likely as result of *foxm1* sequence divergence in relation to other fox genes [62,63]. For the scope of this work, we decided to proceed using the validated sgRNA 8.2.

To understand the nature of mutations generated by sgRNA 8.2., we used TIDE [48], an online tool that identifies and determines the frequency of CRISPR/Cas9-mediated mutations. We found that the most frequent indels generated upon injection of sgRNA 8.2 were deletions of three and nine nucleotides, as detected in 24 hpf F0 embryos (Figure 1B). Embryos injected with sgRNA 8.2 were reared to adulthood and outcrossed. From 14 outcrossed F0 fish, 9 fish (64%) transmitted a mutation in exon 8 of *foxm1* to their offspring (Table 1). We sequenced the target region of these F1 embryos, and we were able to verify the existence of three different mutations, corresponding to the deletion of 3, 9 or 12 nucleotides (Table 1). Strikingly, in all analyzed F1 animals, no frameshift mutations were detected. Although recent studies have shown that Cas9-induced DNA breaks and repair outcomes are non-random [64,65], we expect that about two in three indels would result in a frameshift mutation, as an even distribution of frames resulting from indels was expected [46,53]. We analyzed the results reported by Hwang and colleagues [46] in nine loci, and we found that 72.7% of the reported mutant sequences corresponded to frameshift-inducing indels. Applying a Fisher’s exact test, we found that the frequency of frameshift mutations observed in the 14 mutations described in Table 1 is statistically different from what has been previously described (80 frameshift mutations out of 110, vs. 0 frameshift mutations out of 14; *p* < 0.0001). Therefore, the total absence of frameshift-inducing indels further suggests that frameshifts in the *foxm1* gene targeted regions are strongly deleterious to all somatic and germline cells, even in heterozygosity. This strong effect could be associated with a dominant negative effect of the resulting protein, similarly to a known truncated form of the human FOXM1 [19]. To bypass the detrimental effect of *foxm1* targeting with sgRNA 8.2 during embryogenesis and assess the effects of *foxm1* disturbance in non-proliferative cells we next focused on a conditional cell type-specific loss-of-function of *foxm1*.

### 3.2. foxm1 Is Expressed in Skeletal Muscle Cells

**Figure 2:**
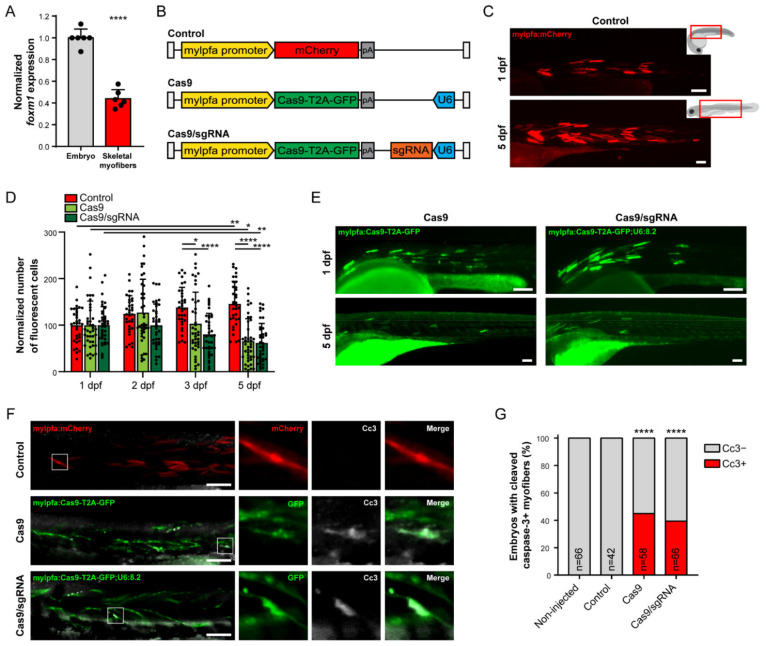
Skeletal muscle cell-specific targeting of *foxm1* with CRISPR/Cas9 is detrimental. (**A**) Expression of *foxm1* in whole zebrafish embryo (except skeletal myofibers) and FACS-sorted skeletal myofibers at 3 dpf. Values are mean ± SD from *n* = 6. **** = *p* < 0.0001 by Student’s *t*-test. (**B**) Representation of the Tol2 cassettes used for transgenesis in this study. White bar represents the Tol2 recombination sites, pA refers to the poly-A sequence and U6 refers to the ubiquitous U6 promoter. (**C**) Embryo injected with mylpfa:mCherry, at 1 (top) and 5 dpf (bottom). Fluorescence microscopy images correspond to the embryo areas indicated by red boxes on the right. (**D**) Quantification of mCherry-positive (Control) or the GFP-positive (Cas9 and Cas9/sgRNA) cells at 1, 2, 3 and 5 dpf. Values are mean ± SD from *n* ≥ 34 embryos, * = *p* < 0.05, ** = *p* < 0.01, **** = *p* < 0.0001 by one-way ANOVA with Bonferroni correction for multiple comparisons. (**E**) Embryo injected with mylfpa:Cas9-T2A-GFP (Cas9) and mylfpa:Cas9-T2A-GFP;U6:8.2 (Cas9/sgRNA), at 1 (top) and 5 dpf (bottom). (**F**) 2 dpf embryos injected with mylpfa:mCherry (Control), mylfpa:Cas9-T2A-GFP (Cas9) or mylfpa:Cas9-T2A-GFP;U6:8.2 (Cas9/sgRNA) stained with anti-cleaved caspase-3 (Cc3) antibody. (**G**) Quantification of 2 dpf embryos with Cc3-negative (Cc3−, gray) and Cc3-positive (Cc3+, red) myofibers from *n* ≥ 42 embryos per condition. **** *p* < 0.0001 by Fisher’s exact test using the discrete number of embryos per condition, comparison with control embryos. Scale bar: 100 µm.

Considering the reported roles of *foxm1* in biological processes beyond the cell cycle [3,20,21], we studied the phenotype generated by the loss of *foxm1* in skeletal muscle cells. Although skeletal muscle cells keep their cell cycle machinery intact [66], the study of skeletal myofibers in zebrafish somites, which are fully differentiated and do not proliferate in response to injury [27], allowed us to investigate for potential roles of *foxm1* beyond cell cycle regulation. We started by assessing whether *foxm1* was indeed expressed in muscle fibers. We used a previously established line that expresses DsRed in the fast-twitching muscle fibers [67] and measured *foxm1* expression in FACS-sorted DsRed-positive cells, in 3 dpf embryos. When compared to the DsRed-negative embryo cells, that include all kind of proliferative and differentiated cells except fast-twitching muscle cells, skeletal muscle cells expressed *foxm1* at lower but detectable levels (Figure 2A). Previous studies analyzing young adult mice by RNA-seq revealed very low but detectable levels of *Foxm1* in single myofibers [35], consistent with our results. Similarly, other studies have shown that purified *Pax7*-negative mouse myofibers also expressed *Foxm1* at low levels [33]. Finally, transcriptomic and proteomic data from the Human Protein Atlas [68] further show that *FOXM1* is very mildly expressed in human skeletal muscle. These data show that three phylogenetically distant vertebrates, zebrafish, mouse and human, express *foxm1* in muscle fibers, suggesting a conserved role, likely in cell homeostasis.

### 3.3. foxm1 Loss-of-Function and Strong Cas9 Expression Impair Muscle Cell Viability

To track individual muscle fibers in time, we built a Tol2 transposon carrying a cassette with the *mylpfa* promoter driving expression of mCherry (mylpfa:mCherry, Figure 2B,C). The myosin light chain, phosphorylatable, fast skeletal muscle a (*mylpfa*) gene is specific to fast-twitch cells, and its cell-specific promoter has been frequently used to create zebrafish transgenic reporter lines and study fast-twitching muscle cells in development [69], myogenesis [70] and regeneration [71]. The gene is active in somitic muscle as well as eye, jaw, gill and fin muscles [69]. We quantified the mCherry-positive cells in the somitic skeletal muscle of individual embryos at 1, 2, 3 and 5 dpf and observed that the number of mCherry-positive muscle fibers increased significantly between 1 and 5 dpf (Figure 2C,D and Figure S2A). This result translates the known formation of new myofibers from satellite cells and progenitors throughout the continuous growth of skeletal muscle in zebrafish [40].

Next, we performed a conditional loss-of-function of *foxm1*, targeting specifically fast-twitching muscle fibers. We used a previously described modular cell type-specific CRISPR/Cas9-mediated mutagenesis system [40]. Using this system, we have generated a Tol2 transposable element containing a cassette with the *mylpfa* promoter driving expression of Cas9-T2A-GFP and the ubiquitous promoter U6 driving expression of the sgRNA 8.2 (mylfpa:Cas9-T2A-GFP;U6:8.2, Figure 2B). To account for possible Cas9 effects, we used the same cassette without sgRNA as control (mylfpa:Cas9-T2A-GFP) (Figure 2B). We injected both constructs in parallel and found that the number of GFP-positive cells decreased significantly at 3 dpf and onwards, including upon Cas9 expression alone (Figure 2D,E and Figure S2A). This result suggests that strong expression of Cas9 in muscle fibers is enough to induce cell toxicity and clearance. Interestingly, we observed that the co-expression of sgRNA and Cas9 reduced the number of GFP-positive cells early on at 2 dpf and induced a highly significant decrease from 3 dpf onwards, in comparison to Cas9 expression alone that had a more modest initial impact and reduced the number of cells only from 3 dpf onward (Figure 2D). To validate the cell type-specific CRISPR/Cas9-mediated mutagenesis system, we performed DNA extraction from 2dpf whole embryos injected with mylfpa:Cas9-T2A-GFP;U6:8.2, and FACS-sorted GFP-positive cells derived from those embryos, and we were able to found indels in the targeted *foxm1* locus (Figure S2B,C). Thus, although Cas9 is enough to induce a decrease in cell number, the formation of indels in exon 8 of *foxm1* in muscle fibers is slightly more impactful, pointing to a *foxm1* function in differentiated non-proliferative fast-twitching muscle fibers. One possibility is the *foxm1* role in DNA damage response [20] that might counteract the cell damage induced by Cas9 overexpression. Another possibility could be the recently described *foxm1* role in the regulation of mitochondrial respiration [21], critical for muscle cell viability. Interestingly, this novel *foxm1* role in the regulation of mitochondrial functions is independent of transcriptional activity and would apply even if sgRNA 8.2 generates a C-terminal truncated isoform.

The deleterious effect of the strong, continuous expression of Cas9 alone, driven by the potent *mylpfa* promoter, might be caused by accumulation of high levels of the protein, affecting proteostasis, and by random DNA nicks and DSBs in unspecific sites at a scale that cells are unable to cope with and thus senesce or die. We investigated the fate of Cas9- and Cas9/sgRNA-expressing cells by performing an immunostaining for cleaved caspase-3 (Cc3), a main effector of apoptotic cell death [72,73]. Caspase-3 is conserved in zebrafish [74] and Cc3 has been successfully detected in zebrafish embryos [50,75,76]. We injected the mylpfa:mCherry (Control), mylfpa:Cas9-T2A-GFP (Cas9) and mylfpa:Cas9-T2A-GFP;U6:8.2 (Cas9/sgRNA) vectors and quantified the number of 2 dpf embryos with Cc3-positive myofibers in each condition (Figure 2F,G). No Cc3-positive myofibers were detected in non-injected and control embryos, suggesting that the integration of the Tol2 transposon and expression of the reporter gene do not cause apoptosis. In contrast, expression of Cas9 in myofibers induces muscle cell death in a significative number of embryos. The number of Cc3-positive cells per embryos was low (mean = 1.02 cells/embryo), in agreement with Cc3-positive dying cells being efficiently cleared by the zebrafish immune system’s phagocytes [77,78], and with the decreasing number of Cas9-T2A-GFP-expressing myofibers observed in time. Coexpression of sgRNA 8.2 in myofibers generated equivalent low number of Cc3-positive cells per embryo (mean = 1.03 cells/embryo), again consistently with efficient clearance and observed reduction in GFP-positive muscle cells in time. Thus, our data suggest that *mylpfa*-driven Cas9 expression in myofibers is detrimental to cell homeostasis and causes apoptosis. This raises safety concerns about the use of Cas9 in in vivo models and to clinical applications. Interestingly, recent data suggests that Cas9 induces DNA DSBs, genomic instability, and cell cycle arrest independently of sgRNAs binding or even nuclease activity [42], and in vivo studies in mice suggest Cas9 may be neurotoxic [79].

### 3.4. foxm1 in Muscle Cells Contributes to Non-Autonomous Tissue Repair

**Figure 3:**
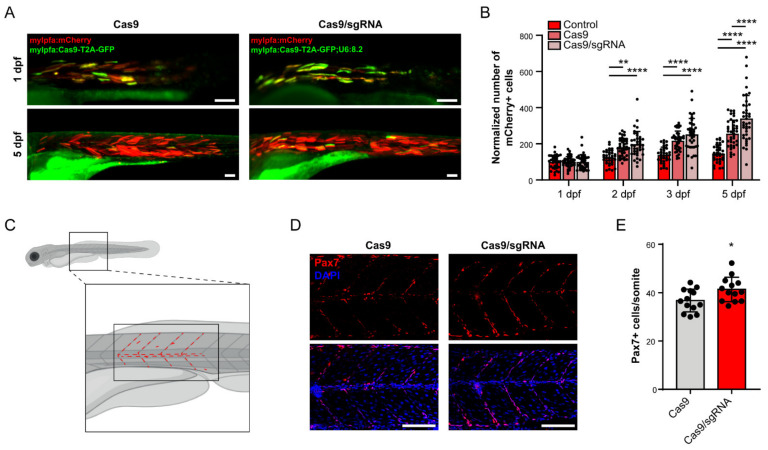
*foxm1* in skeletal muscle cells contributes for non-autonomous signaling and tissue homeostasis. (**A**) mCherry-positive cells in embryos also injected with mylfpa:Cas9-T2A-GFP (Cas9) and mylfpa:Cas9-T2A-GFP;U6:8.2 (Cas9/sgRNA), at 1 and 5 dpf. (**B**) Quantification of mCherry-positive cells in control, Cas9 and Cas9/sgRNA embryos at 1, 2, 3 and 5 dpf. Values are mean ± SD from n ≥ 34 embryos per condition. ** *p* < 0.01, **** *p* < 0.0001 by one-way ANOVA with Bonferroni correction for multiple comparisons. (**C**) Illustration of a 3 dpf larva and the region used for Pax7 immunostaining quantitative analysis. Red dashed lines represent the quantified cells in the central myotome and the vertical and horizontal myosepta. (**D**) Embryos of 3 dpf injected with mylfpa:Cas9-T2A-GFP (Cas9) or mylfpa:Cas9-T2A-GFP;U6:8.2 (Cas9/sgRNA) stained with anti-Pax7 antibody and DAPI. (**E**) Quantification of Pax7-positive cells per somite in 3 dpf embryos. Values are mean ± SD, from *n* = 13 embryos per condition. * = *p* < 0.05 by a Student’s *t*-test. Scale bar: 100 µm.

Previous data has shown that manipulations of *FOXM1* expression in human primary dermal fibroblasts affect clusters of genes associated with cell senescence and cell communication, namely through regulation of senescence-associated secretory phenotype (SASP) genes [3]. Interestingly, in recent years several studies have established the association of myokines secreted by muscle fibers with paracrine, autocrine and endocrine effects with systemic and local impact [80,81,82], such as muscle homeostasis and remodeling itself, namely in zebrafish [83,84]. We therefore asked whether *foxm1* loss-of-function in muscle cells has non-autonomous effect in tissue repair. Since a decrease in cell number and increase in apoptosis was clear upon injection of the mylfpa:Cas9-T2A-GFP;U68.2 and mylfpa:Cas9-T2A-GFP vectors, tissue regeneration is expected to occur and revert cell loss. Supporting this hypothesis, increased number of dead embryos was not observed in these two conditions, thus suggesting that active homeostatic mechanisms are likely compensating cell loss. Therefore, we further explored how the tissue was responding to the damage in myofibers. For that, we coinjected the mylpfa:mCherry cassette and the mylpfa:Cas9-T2A-Cas9 cassette with and without the sgRNA 8.2 and quantified the number of mCherry- and GFP-positive cells at 1, 2, 3 and 5 dpf (Figure 2B, Figure 3A,B and Figure S2A). In this experiment, by counting the variation of mCherry-positive cells, we were able to assess the muscle-specific response to the damage caused by Cas9 alone or Cas9 and *foxm1* indels combined. We observed that the increase in the number of mCherry-positive cells was more pronounced in the coinjection conditions, supporting the idea that cell damage caused by Cas9 toxicity induced non-autonomous cell response toward muscle fiber regeneration (Figure 3A,B). Embryos injected with the cassette containing the sgRNA 8.2 presented significantly more mCherry-positive cells than the embryos injected with the mylpfa:Cas9-T2A-GFP cassette at 5 dpf (Figure 3A,B). Therefore, *foxm1* loss-of-function specifically in fast-twitching muscle fibers significantly increases myogenesis in response to damage, suggesting that *foxm1* is required for proper tissue homeostasis. Critically, we did not observe a significant difference in the number of GFP-positive cells between embryos injected with or without the sgRNA-containing vector (Figure 2D), neither a significant difference in the number of embryos with Cc3-positive myofibers, excluding a causal contribution of differences in cell loss for the differences in mCherry-positive cells observed between Cas9 alone and combined with *foxm1* indels.

To further investigate the cell non-autonomous effect of *foxm1* in myogenesis, we quantified the number of Pax7-positive satellite cells in the central myotome and the vertical and horizontal myosepta in 3 dpf embryos injected with the mylfpa:Cas9-T2A-GFP;U6:8.2 or mylpfa:Cas9-T2A-Cas9 cassettes (Figure 3C). The activation and proliferation of these muscle-resident stem cells occurs during larval and adult tissue growth and regeneration [25,27,32,85]. We found a significant increase in the number of Pax7-positive cells per somite in the larvae treated with the sgRNA 8.2 (Figure 3D,E). Previous data has shown an increase in the number of Pax7-positive satellite cells in response to small muscle injury, in zebrafish embryos with 3 dpf [85,86], suggesting that our genetic manipulation of the *foxm1* locus generates a similar non-autonomous signal that also stimulates satellite cells activation and proliferation, followed by differentiation into myoblasts and formation of new myofibers. Further studies are needed to elucidate the specific signaling pathways involved in the paracrine effect being affected by *foxm1* manipulation. It is known that mammalian FoxM1 regulates expression of cytokines such as IL-6 [3,87,88], a myokine produced by muscle fibers [89,90] that has been associated with satellite cell and myoblast proliferation and differentiation via the JAK-STAT pathway [90] in humans. Other growth factors and cytokines, such as leukemia inhibitory factor (LIF) [91], hepatocyte growth factor (HGF) [92], bone morphogenetic proteins (BMPs) [93,94,95] and fibroblast growth factors (FGFs) [96] have also been associated with satellite cell proliferation and differentiation.

In sum, our results suggest that normal *foxm1* expression in differentiated skeletal muscle fibers contributes to tissue homeostasis in response to cell death. Considering the beforementioned role of *foxm1* in the modulation of cytokines expression and in the inhibition of SASP, and the signaling pathways impacting satellite cells, our data supports the contribution of *foxm1* to a non-autonomous regulation of satellite cells activation and formation of new muscle fibers in response to cell injury. This regulation may prevent stem cell exhaustion and deregulation of tissue size. Although further studies on the genes being regulated by *foxm1* in myofibers and the associated mechanisms of satellite cell activation are needed, our results add evidence to the role for *foxm1* as a regulator of cell signaling genes [3,97,98], controlling non-autonomous stem cell activation and proliferation in response to tissue damage.

## 4. Conclusions

Our work points to a role of *foxm1* in zebrafish development and fast-twitching muscle cell homeostasis. Using CRISPR/Cas9 technology with a single sgRNA we generated several stable mutant lines with small deletions in the transactivation domain of FoxM1. The lack of frameshift mutations in somatic cells and germ line transmission suggests loss of *foxm1* expression is strongly deleterious.

We also reported that expression of Cas9 by a strong, cell-specific promoter, *mylpfa*, leads to cell death. Moreover, Cas9-associated cell loss seems to be accelerated by CRISPR/Cas9-mediated mutagenesis of the *foxm1* gene, supporting a cell autonomous function in muscle cells. Crucially, our results also suggest that the muscle cell-specific loss of *foxm1* contributes to satellite cell activation and proliferation, evidencing an additional cell non-autonomous role of *foxm1* required for skeletal muscle homeostasis.

## Figures and Tables

**Table 1:** Genomic deletions driven by sgRNA 8.2, protein translation and respective frequencies of germline transmission detected in F1 embryos.

Genotype	DNA Sequence	aa Sequence	Frequency (*N*)
Wild type	…AAGATGAAGCCTCTACTGCCTCGGACTGAC…	…KMKPLLPRTD…	36% (5)
3 nts deletion	…AAGATGAAGCCTCTACTGCCTCGGACTGAC…	…KMKPLLPRTD…	7% (1)
9 nts deletion	…AAGATGAAGCCTCTACTGCCTCGGACTGAC…	…KMKPLLPRTD…	43% (6)
12 nts deletion	…AAGATGAAGCCTCTACTGCCTCGGACTGAC…	…KMKPLLPRTD…	14% (2)

nts, nucleotides; aa, amino acid; N, number of embryos.

## Supplementary Materials

The following are available online at https://www.mdpi.com/article/10.3390/cells10051241/s1, Figure S1: Validation of CRISPR/Cas9-mediated gene disruption methodology, detection of heteroduplexes and impact of sgRNA 8.2 in F0 embryos. Figure S2: Representative images of zebrafish embryos from the different conditions through time, at 1, 2, 3 and 5 dpf and cell-specific impact of sgRNA 8.2 in 2 dpf myofibers. Table S1: Oligonucleotides used for targeting *foxm1*. Target-specific sequence in capital letters. Overhangs for insertion into expression vector in lowercase letters. Table S2: Primers used in this study. Table S3: Primers used for RT-qPCR analysis.
