# Supplementary material for: foxm1 Modulates Cell Non-Autonomous Response in Zebrafish Skeletal Muscle Homeostasis
Source: Cells. 2021 May 18;10(5):1241. doi: 10.3390/cells10051241 (PMC8158134; doi:10.3390/cells10051241)
Supplement: Supplementary file 1 [file cells-10-01241-s001.zip › Supplementary Figures_revision.pdf]

Supplementary Figures

Figure S1

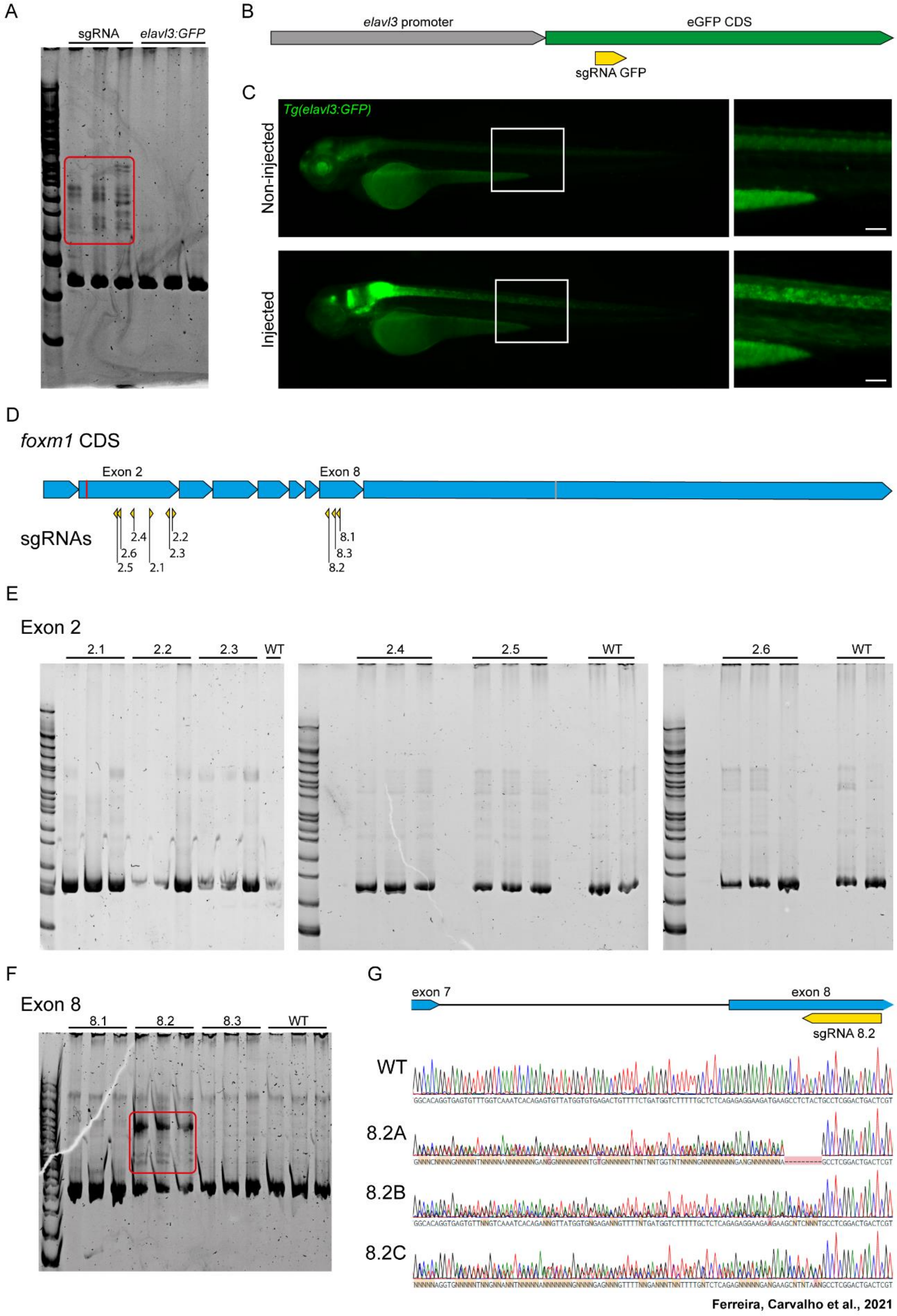

Figure S1. Validation of CRISPR/Cas9-mediated gene disruption methodology, detection of heteroduplexes and impact of sgRNA 8.2 in F0 embryos. (**A**) Polyacrylamide gel electrophoresis (PAGE) showing the amplicon of the eGFP coding sequence (CDS) of three batches of F0 *Tg(elavl3:GFP)* injected (sgRNA) and non-injected control (*elavl3:GFP*) embryos, both 24 hpf, in which it is possible to detect multiple heteroduplexes (red square) that resulted from CRISPR/Cas9-mediated mutagenesis. (**B**) Representation of the *elavl3:GFP* reporter cassette targeted by CRISPR/Cas9. (**C**) Representative images of *Tg(elavl3:GFP)* embryos, 72 hpf, non-injected (top) or injected with the Cas9 mRNA and sgRNA cocktail (bottom). The white rectangule and corresponding magnification insets on the right show the cell mosaicism resulting from this approach and reflect the efficacy of CRISPR/Cas9-mediated on eGFP expression. (**D**) Representation of the *foxm1* CDS, showing exon-exon borders, and the localization of the sgRNAs used in this study. The red bar in exon 2 represents the first translated codon. (**E**) PAGE showing the amplicon of the genomic regions in exon 2 of *foxm1* targeted with sgRNAs 2.1 to 2.6 and non-injected sibling controls, from batches of 24 hpf embryos. (**F**) PAGE showing the amplicon of the genomic region in exon 8 of *foxm1* targeted with sgRNAs 8.1 to 8.3 and non-injected sibling controls, from batches of 24 hpf embryos. It is possible to detect heteroduplexes (red square) that resulted from CRISPR/Cas9-mediated mutagenesis using sgRNA 8.2. (**G**) Representation of the *foxm1* locus targeted with sgRNA 8.2 (top) and Sanger sequencing chromatograms of that same locus (bottom) from one batch of non-injected (WT) and three heteroduplexes bands isolated with PAGE from injected batches (8.2A, 8.2B and 8.2C) of 24 hpf embryos. Scale bar: 100 µm.

Figure S2

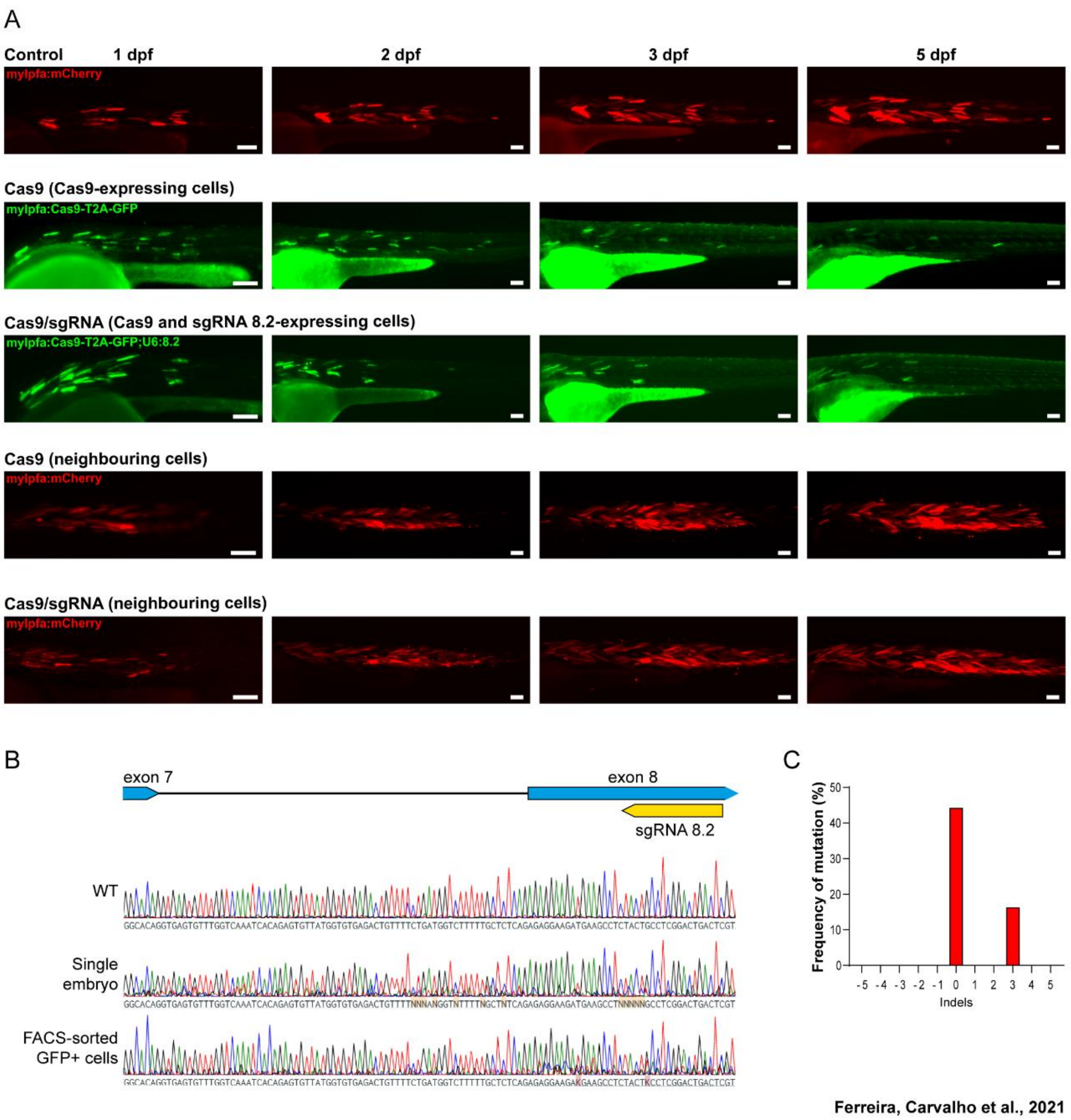

Figure S2. Representative images of zebrafish embryos from the different conditions through time, at 1, 2, 3 and 5 dpf and cell-specific genomic editing by sgRNA 8.2 in 2 dpf myofibers. (**A**) One-cell stage embryos were injected with the mylpfa:mCherry cassette alone (Control) or the mylpfa:mCherry cassette together with either the mylpfa:Cas9-T2A-GFP (Cas9) or mylpfa:Cas9-T2A-GFP;U6:8.2 (Cas9/sgRNA) cassettes. The two bottom rows show mCherry-positive cells in embryos also injected with the GFP-carrying cassette. (**B**) Representation of the *foxm1* locus targeted with sgRNA 8.2 (top) and Sanger sequencing chromatograms of that same locus (bottom) from one non-injected embryo (WT), from an heteroduplex band isolated with PAGE from a single embryo injected with mylpfa:Cas9-T2A-GFP;U6:8.2 (Single embryo), and from FACS-sorted GFP-positive cells from a batch of embryos injected with mylpfa:Cas9-T2A-GFP;U6:8.2 (FACS-sorted GFP+ cells). (**C**) Statistically significant indels detected in a 2 dpf embryo

injected with mylpfa:Cas9-T2A-GFP;U6:8.2. Frequency reflects abundancy of the WT and mutant alleles from an heteroduplex band isolated with PAGE from a single embryo. Scale bar: 100 µm.
