# Supplementary material for: foxm1 Modulates Cell Non-Autonomous Response in Zebrafish Skeletal Muscle Homeostasis
Source: Cells. 2021 May 18;10(5):1241. doi: 10.3390/cells10051241 (PMC8158134; doi:10.3390/cells10051241)
Supplement: Supplementary file 1 [file cells-10-01241-s001.zip › Supplementary Tables_revision.pdf]

Supplementary Tables

Table S1. Oligonucleotides used for *foxm1* targeting. Target-specific sequence in capital letters. Overhangs for insertion into expression vector in lowercase letters.

Expression vector	Name	Sequence (5'-3')	
pDR274	2.1	fw	taggGGAAGGAGTGTGGGCCTC
		rv	aaacGAGGCCCACACTCCTTCC
	2.2	fw	taggTGTTTTTCTACAGAACTT
		rv	aaacAAGTTCTGTAGAAAAACA
	2.3	fw	taggTTTTGCTCTCCTCCAAAC
		rv	aaacGTTTGGAGGAGAGCAAAA
	2.4	fw	taggGTGTCCGGCATAGTAGGG
		rv	aaacCCCTACTATGCCGGACAC
	2.5	fw	taggGTCGAGGTGGTCTTACAG
		rv	aaacCTGTAAGACCACCTCGAC
	2.6	fw	taggGAGCAGTGGACTGGGTCG
		rv	aaacCGACCCAGTCCACTGCTC
	8.1	fw	taggGAACAGTGACTGACCCAG
		rv	aaacCTGGGTCAGTCACTGTTC
	8.2	fw	taggGTCAGTCCGAGGCAGTAG
		rv	aaacCTACTGCCTCGGACTGAC
	8.3	fw	taggGGAAGCTGAATGGGTACC
		rv	aaacGGTACCCATTCAGCTTCC
	GFP	fw	taggAGAGACCGAGAGAGGGTCTCA
		rv	aaacTGAGACCCTCTCTCGGTCTCT
pDestTol2pA2-U6:gRNA	8.2	fw	GTCAGTCCGAGGCAGTAGgt
		rv	CTACTGCCTCGGACTGACga

Table S2. Primers used in this study.

Target locus	Sequence (5'-3')	
elavl3 promoter	fw	AGTGATGATGTATAGAGTGATGTTTACG
	rv	CTTCGATTTGCAGGTCTTCAGGC
sgRNA 2.1-2.3	fw	CACCCTACTATGCCGGACAC
	rv	TAACTCACCTTTCCCAAGCTC
sgRNA 2.4-2.6	fw	GGGAGAGCCCAAGGAGACC
	rv	GGCGAAACAAGTTCATCCTGC
sgRNA 8.1-8.3	fw	AAAGCCATACCTGCATTGGGTG
	rv	TCATCACTTGCTTTGCCAGAATG
sgRNA GFP	fw	GGTGGTGCCCATCCTGG
	rv	GCACGCCGTAGGTCAGG

Table S3. Primers used for RT-qPCR analysis.

**Target gene**	**Sequence (5'-3')**	
foxm1	fw	AGCAAGAGGGTCCGCATCG
	rv	GGTCACAGGCTGAAACACAGG
b2m	fw	AGGATTGTCTGCTTGGCTCTCT
	rv	GGAGTGGAGACTTTCCCCTGTAC
eef1a1	fw	CTTCTCAGGCTGACTGTGC
	rv	CCGCTAGCATTACCCTCC
